# Insights into Essential Oil and Their Electroactive Constituents: Recent Progress and Challenges in Electro-Sensing Strategies for Food Analysis

**DOI:** 10.3390/molecules31132214

**Published:** 2026-06-24

**Authors:** Mihaela Buleandră, Dana Elena Popa, Eliza Oprea, Irinel Adriana Badea, Anca-Daniela Raiciu

**Affiliations:** 1 Department of Analytical Chemistry and Physical Chemistry, University of Bucharest, 90–92 Panduri, 050663 Bucharest, Romania; irinel.badea@chimie.unibuc.ro; 2 Department of Botany and Microbiology, Faculty of Biology, University of Bucharest, 1–3 Portocalilor, 060101 Bucharest, Romania; eliza.oprea@g.unibuc.ro; 3 Department of Pharmacognosy, Phytochemistry and Phytotherapy, Faculty of Pharmacy, Titu Maiorescu University, 16 Gheorghe Sincai, 040314 Bucharest, Romania; daniela_raiciu@yahoo.com

**Keywords:** essential oils, electroanalytical methods, volatile compounds, antioxidant properties

## Abstract

Essential oils are extracted from various parts of plants and have many beneficial properties and applications. These include aromatherapy, healthcare, cosmetics, fragrances, agriculture, household cleaning products, and the food industry. Due to their antimicrobial and antioxidant properties, essential oils are suitable for use as natural flavorings and preservatives, ensuring food quality maintenance and facilitating clean-label product production. In this context, assessing the quality of essential oils is of paramount importance. Among the various analytical methods, electrochemical methods stand out for their simplicity, cost-effectiveness, and environmental friendliness. Consequently, this review examines the applications, advantages, disadvantages, and limitations of electroanalytical methods proposed to quantify major volatile, electroactive components and determine their antioxidant properties. The objective of this evaluation is to establish a framework for future research that will address existing gaps and shortcomings in electroanalytical methodologies.

## 1. Introduction

There are numerous explanations for the term “essential oils”, but the International Organization for Standardization (ISO) provides the following definition: products extracted from natural, plant-based raw materials through steam distillation, mechanical processing of citrus peels, or dry distillation [1]. These oils are mixtures of volatile and lipophilic compounds with an intense and characteristic smell and taste, synthesized by aromatic plants as secondary metabolites [2]. It is estimated that around 3000 essential oils have been isolated from various plant species, but only about 300 of these are used commercially [3].

In general, essential oils represent less than 5% of dry plant matter. The number of their volatile constituents typically ranges from 20 to 60 [4], though it can exceed 300 in some cases, with highly variable concentrations [5]. Sometimes, one major component dominates the oil’s composition, accounting for up to 95% of it. In other cases, several components together constitute more than half of the total volatile compound content. Trace components represent less than 1% of the essential oil content. The proportions of constituents in essential oils vary greatly depending on the following factors: plant species, variety, part of the aromatic plant that is used (e.g., leaves, flowers, fruits, seeds, roots, rhizomes, wood, or bark), harvest time, crop location, growing conditions, and extraction method [6].

The aroma and properties of an essential oil result from the combination of all its components, including minor constituents. While the most abundant substances are indeed the key to an essential oil’s overall aroma and biological and therapeutic activities, trace components can also play a role in imparting unique characteristics, either by enhancing the effects of major components or by producing antagonistic or additive outcomes [7].

**Figure 1:**
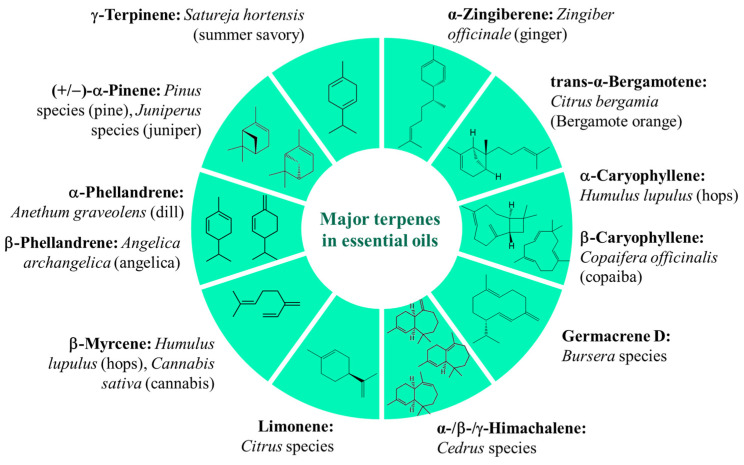
Major terpene constituents of essential oils.

Essential oils are primarily composed of volatile organic compounds fitting structurally to terpenes and their oxygenated derivatives (terpenoids), as well as non-terpenic compounds. According to the isoprene rule proposed by Otto Wallach, terpenes are characterized by a molecular structure consisting of isoprene units; the structural diversity of terpenes is mainly determined by the number and arrangement of these building blocks. Monoterpenes and sesquiterpenes are common terpenes found in essential oils as major components (Figure 1).

**Figure 2:**
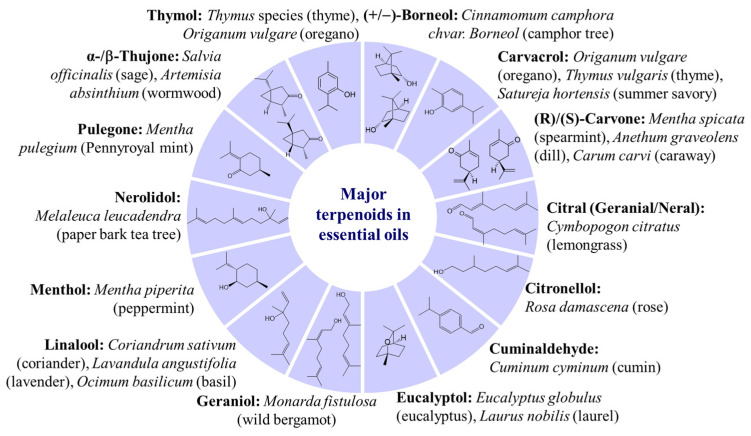
Major terpenoid constituents of essential oils.

Terpenoids are terpene compounds that possess oxygen-containing functional groups as a result of biochemical transformations. Figure 2 illustrates the most prevalent terpenoids found in essential oils.

**Figure 3:**
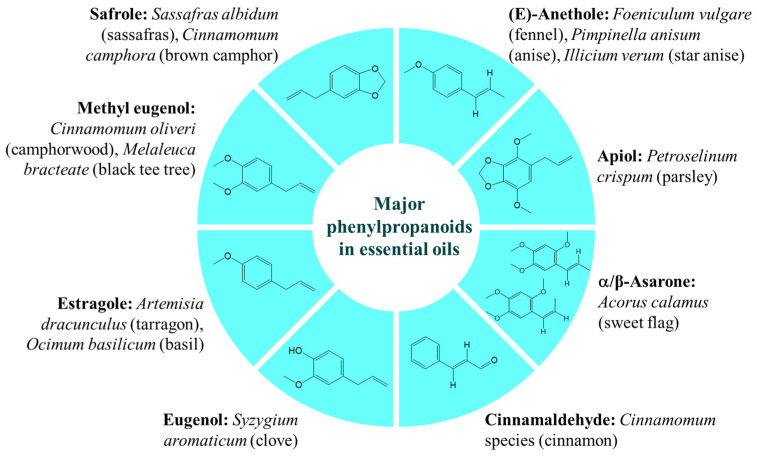
Major phenylpropanoid constituents of essential oils.

Terpenes and terpenoids are the largest group present in essential oils, often making up around 90% of their composition. However, certain plant species also contain phenylpropanoid compounds, which are sometimes the main component. Figure 3 shows the structures of these compounds and the essential oils in which they are found as primary constituents.

**Figure 4:**
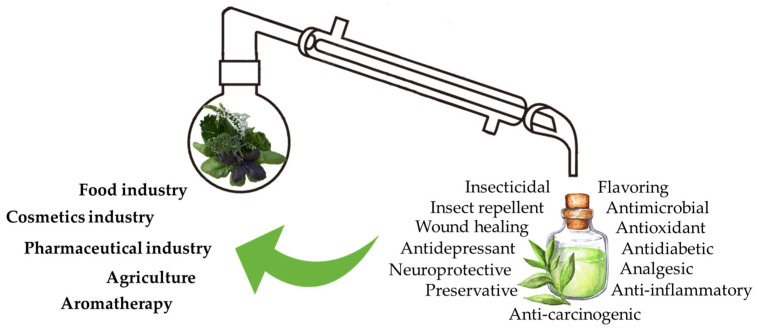
Biological activities and applications of essential oils.

Essential oils also include non-terpenic components, such as straight-chain compounds like alcohols, aldehydes, ketones, acids, and esters, as well as sulfur- and nitrogen-containing compounds [8]. These include benzaldehyde, which is a major component in the leaves of *Prunus* species, and benzyl benzoate, found in significant amounts in Peru balsam and certain species of cinnamon. Essential oils are versatile and have countless applications, including as food preservatives, flavoring agents, fragrances, pharmaceuticals, and therapeutic agents (Figure 4). In addition to the uses mentioned above, essential oils have utilizations in agriculture as biopesticides, as well as in animal husbandry [9].

These natural essences are highly valued for their distinctive taste. For example, cinnamon, mint, lavender, and various citrus oils are frequently used to flavor desserts, chocolate, candy, cookies, and milk. Other oils, such as thyme, basil, oregano, sage, and rosemary, are more suitable for savory dishes. Essential oils act as preservatives, too. Thus, they inhibit the growth of harmful microorganisms such as bacteria, viruses, and fungi. Also, thanks to their antioxidant properties, they prevent oxidation, preserving the freshness and nutritional value of food. These characteristics make them ideal for extending the shelf life of food products. Thanks to all these properties, essential oils are increasingly used in the food industry, both as natural flavorings and as preservatives [10].

Natural essential oils are currently an alternative for use in food or packaging, especially after the identification of adverse effects of synthetic antioxidants such as butylated hydroxyanisole (BHA), butylated hydroxytoluene (BHT), and tert-butylhydroquinone (TBHQ) [11]. Although the effectiveness of natural antioxidants and their flavor can sometimes be questionable, adding essential oils to food aligns with the increasing consumer demand for clean-label products.

An assessment of the global market showed that in 2025 the global market was worth USD 13.66 billion, with Europe dominating 43.36% of the total [12]. Orange and lemon oils are the most abundant, followed by eucalyptus, lavender, rosemary, tea tree, and peppermint. Estimates show that between 2026 and 2034, this market will have a compound annual growth rate (CAGR) of 11.08%, with both food and beverage applications segment accounting for approximately 40%.

In food the occurrence of essential oils is regulated by Regulation (EC) No. 1334/2008 on flavorings [13], which covers, among other things, safety and labeling requirements for products in order to protect consumers. This regulation includes a list of flavorings and raw materials approved for use in food products and specifies which flavors require evaluation and approval by the European Food Safety Authority (EFSA). It also imposes the maximum concentrations for certain natural substances and prohibits the addition of others. In the US, the Food and Drug Administration (FDA) publishes a list of essential oils recognized as GRAS (“Generally Recognized as Safe”) that can be used in food [14]. Regardless of the oil used, good manufacturing practices must be followed.

Although essential oils are generally safe for consumption, their high concentration can be harmful, which is why they should be used in small amounts or diluted. Their lipophilic nature, volatility, and intense aroma can either reduce their effectiveness in foods with a high-water content or affect the taste or smell of products [15]. An effective solution is to incorporate them into packaging materials for controlled release, maintaining biological activity and minimizing the impact on sensory characteristics [16].

However, the high value of essential oils encourages adulteration, through the use of oils from different regions, related species, synthetic compounds, or dilution with vegetable oils, which can reduce their effectiveness and increase the risk of allergic reactions [17].

For these reasons, it is essential to develop efficient analytical methods able to guarantee the quality control of essential oils and the correct identification of their components. Techniques such as GC-MS, GC-FID, chiral gas chromatography, and isotope ratio mass spectrometry (IRMS) allow the analysis of chemical composition and enantiomeric ratios. Spectroscopic techniques, such as infrared spectroscopy and Raman spectroscopy, combined with chemometrics, facilitate rapid screening and quality control, providing detailed information about the structure of molecules [17,18]. Compared to these methods, which are costly and require expertise, electrochemical techniques are an affordable, simple, and portable alternative with low solvent consumption and minimal waste generation. In addition, they reduce the laborious sampling steps, preventing the degradation of active compounds due to volatility.

The review provides a brief overview of the antimicrobial and antioxidant properties that support the use of essential oils in the food industry. However, the main focus is on understanding the current state and future directions of electrochemical sensors used to detect and quantify the constituents of the essential oils and to determine their antioxidant properties.

## 2. The Relevance of the Antioxidant and Antimicrobial Properties of Essential Oils in Food Systems

Essential oils contain several compounds with recognized antioxidant activity, particularly phenolic constituents such as eugenol, methyl eugenol, thymol, carvacrol, and cinnamaldehyde [19,20,21]. Other important antioxidant phenylpropanoids include trans-anethole, estragole, β-asarone, safrole, apiol, and vanillin [19,22,23,24,25,26]. However, some of these compounds, particularly β-asarone, estragole, methyl eugenol, pulegone, safrole, and thujones, have been associated with toxic, mutagenic, carcinogenic, or neurotoxic effects and are therefore regulated in foods by Regulation (EC) No. 1334/2008 [13]. Diverse antioxidant essential oils originate from citrus fruits (limonene, α-pinene, β-pinene), lemongrass (citral, citronellal), peppermint (menthone, menthol), and spearmint (limonene, carvone) [27,28].

These molecules can scavenge reactive oxygen species and interrupt oxidative chain reactions, thereby contributing to food preservation and shelf-life extension. Because the antioxidant efficacy of essential oils strongly depends on the identity and concentration of these bioactive constituents, their accurate determination is of considerable interest in food analysis.

Antioxidant activity is commonly assessed using methods such as DPPH, ABTS, FRAP, and ORAC, alongside emerging analytical technologies including nanomaterial-based sensors and microfluidic platforms [29]. In addition, many antioxidant constituents of essential oils are electroactive molecules with phenolic (eugenol, thymol, carvacrol) or aldehydic (e.g., citral, citronellal) functional groups that undergo well-defined redox processes. Consequently, they represent important analytical targets for electrochemical sensing platforms developed for quality control, authenticity assessment, and monitoring of essential-oil-based food additives and packaging systems.

On the other hand, essential oils possess well-documented antimicrobial properties against a broad range of foodborne bacteria, fungi, and yeasts. Their activity is mainly attributed to components such as eugenol, carvacrol, thymol, cinnamaldehyde, citral, and various terpene alcohols, which can disrupt microbial cell membranes and interfere with some biochemical processes [30]. Due to these properties, essential oils are increasingly used as natural preservatives in foods and active packaging systems to inhibit microbial growth and extend shelf life.

Some antimicrobial constituents of essential oils have also been reported to induce oxidative stress in microbial cells [31]. The redox-active nature of these compounds is particularly relevant for electrochemical food analysis, as it provides the basis for their sensitive electrochemical detection.

From the perspective of electrochemical food analysis, the antimicrobial compounds responsible for these effects are also among the most relevant essential oil constituents targeted for chemical characterization and quality control. Their concentration, stability, and release in food matrices directly influence the efficacy of preservatives, making their selective detection and quantification important analytical objectives. Consequently, electrochemical sensing strategies have attracted growing interest for monitoring these bioactive essential oil components in foods and food-packaging applications.

## 3. Electrochemical Evaluation of Essential Oils

Electrochemical evaluation of essential oils uses electroanalytical techniques to study and characterize their properties, in particular the presence and concentration of certain compounds, as well as their redox behavior and antioxidant potential. At the same time, this approach is a valuable tool for understanding how the components of essential oils interact with biological systems.

The most commonly used electrochemical techniques in this field are voltammetric techniques. These are based on measuring the current response generated in an electrochemical cell when the applied potential is changed. The change in potential induces variations in the concentration of electroactive species at the surface of the working electrode, through redox reactions. The current thus obtained is directly proportional to the concentration of the analyte under investigation. Some types of voltammetric techniques have been reported, such as cyclic voltammetry (CV), linear sweep voltammetry (LSV), differential pulse voltammetry (DPV), square wave voltammetry (SWV). These methods differ in the way the applied potential varies and in the type of information provided, each having specific advantages in terms of sensitivity, signal resolution, and applicability in the analysis of essential oil compounds. CV and LSV are essential for characterizing redox reactions, while DPV and SWV are superior for detecting low concentrations of analytes because the background current is smaller in these cases.

Voltammetric techniques depend on the working electrode material in an electrochemical cell, this being the key challenge in developing electrochemical sensors. Bare electrodes are typically employed for basic electrochemical measurements, but their performance is constrained by the properties of the materials they are made of. In contrast, modified electrodes offer several advantages, especially related to selectivity and sensitivity. For this reason, various carbon nanomaterials, including nanotubes and graphene, as well as metal and metal oxide nanoparticles and polymer films, have been synthesized, characterized and used to modify electrodes. This made it possible to create specific, sensitive sensors that can be operated to determine various constituents in essential oils.

In the manuscript are summarized various performance parameters of the analytical methods for comparison purposes, such as linearity ranges, detection limits and quantification limits. However, it should be noted that such a comparison is generally only relevant and meaningful to a certain extent, given the degree of variability. This is because the aforementioned characteristics were established, determined or calculated in a different context in each individual study (e.g., different electrodes, validation strategies and matrices). Accordingly, these values show how well sensors perform in certain experimental conditions, not their overall efficacy.

On the other hand, it is useful to distinguish between electrochemical studies conducted on genuine essential oils and those performed on related, yet distinct, matrices (powdered spices, pharmaceutical products, toothpaste, honey, seawater). Although the latter do not represent direct applications to essential-oil analysis, they are pertinent for illustrating sensor developing and practical relevance.

### 3.1. Quantification of Major Constituents of Essential Oils

#### 3.1.1. Eugenol

Eugenol is electrochemically active due to its phenolic structure, meaning it can undergo redox reactions at an electrode surface. Electrochemical methods have been proposed for analyzing complex matrices containing eugenol, including spices (e.g., cinnamon, cloves, curry and nutmeg). Electrochemical analysis of spices and herbs requires solid–liquid extraction procedures involving time-consuming sample preparation and the use of multiple reagents. Essential oils obtained through distillation, unlike spices and herbs, are already in the liquid phase, which makes their electrochemical analysis easier to perform. Therefore, the distinctive features of the analytical methods stem from the unique properties of the sample matrices, which introduces a high degree of variability and, at the same time, presents a challenge that must be overcome. Despite being one of the most studied electrochemical compounds, relatively few methods have been developed for eugenol determination in essential oils (Table 1).

As can be observed in Table 1, the most commonly used working electrodes were the GCE and the CPE, which are recognized for their versatile surfaces. CPEs are modified by incorporating chemicals into the paste, while GCEs are typically modified by forming films on their surfaces.

A quantitative determination method with a detection limit of 3.8 μM was developed exploiting the irreversible oxidation peak of eugenol obtained on the surface of a GCE in 0.1 M Triton X100 by CV. Using the surfactant medium to solubilize the water-insoluble compound allowed eugenol quantification in clove and basil essential oils. It also reduced the amount of organic solvent required for analyzing various spices [33].

In most cases, the electrochemical signal of eugenol on carbon-based electrodes exhibits low sensitivity. However, modifying the surface of these electrodes can significantly improve their electrocatalytic properties. This modification reduces the overpotential required for the oxidation reaction and increases the peak current of eugenol, which leads to a significantly increased sensitivity of the analytical method. In this context, optimizing the electrode surface becomes essential for obtaining the best possible electrochemical response. Thus, carbon-based nanomaterials and metal nanoparticles are frequently integrated into or onto the surface of electrodes to improve their performance. The first category includes carbon nanotubes (CNTs), such as single-walled nanotubes (SWCNTs), which offer high sensitivity, and multi-walled nanotubes (MWCNT), which offer mechanical robustness.

The combination of MWCNTs-COOH and electropolymerized polyPGR improved the sensor’s ability to detect eugenol across a broad concentration range of 0.75 to 100 µM, with a detection limit of 0.73 µM. Caboxylated MWCNTs provide a large electroactive surface area and enhance the electron transfer rate, while polymeric film improves the sensitivity and selectivity of the determination. The sensor’s performance was validated by comparing the results with those of an independent method based on spectrophotometry [47].

Other examples of GCE modifiers include CeO_2_ nanoparticles dispersed in cetylpyridinium bromide [38], carbon black (CB) nanoparticles and dihexadecyl hydrogen phosphate (DHB) [39], and a hydroxyapatite-TiO_2_ composite [44].

Nanoparticles of CeO_2_ offer a large specific surface area, good electronic conductivity and enhanced catalytic activity. Meanwhile, surfactants improve the dispersion of nanoparticles, providing a larger active surface area and enabling the solubilisation of hydrophobic eugenol molecules at the electrode surface. Combining the GCE with CeO_2_ and CPB resulted in a 2.8-fold increase in the eugenol oxidation current compared to the GCE, and a 70 mV cathodic shift in the oxidation peak potential [38].

The voltammetry of immobilized microdroplets (VIM) procedure was used to determine the concentration of eugenol in clove oil, eliminating the need for complex sample extraction or separation steps by directly immobilizing the oil sample within the CB-DHB film [39]. The clove sample or eugenol standard solution was homogenized into a CB-DHB suspension and cast directly onto the GCE, after which the solvent was evaporated at room temperature. CB nanoparticles promote the electron transfer and increase the active surface area, while DHB reliably traps clove oil microdroplets by creating a robust, hydrophobic lipid film. Commercial samples of clove oil containing between 14% and 68.3% eugenol were analyzed using the proposed VIM procedure. The results were compared with those obtained using a spectrophotometric method, yielding relative standard deviations (RSD%) of less than 5.0%.

The voltammetric sensor based on a hydroxyapatite (HAP)-TiO_2_ composite-modified GCE has resolved the issue of the overlapping signals of eugenol and ascorbic acid. The HAP-TiO_2_ -modified GCE showed an excellent electro-catalytic activity towards oxidation of both ascorbic acid and eugenol enabling the selective and simultaneous determination of the two antioxidants with good selectivity and high accuracy in real samples, such as lemon, vitamin tablets, clove oil, and an Indian traditional Siddha formulation [44].

#### 3.1.2. Carvacrol and Thymol

Carvacrol and thymol, commonly found in thyme and oregano, are isomeric monoterpenoids with a phenolic structure. Each compound has three functional groups: a hydroxyl group, a methyl group, and an isopropyl group (Figure 2). The key difference between the two lies in the position of the hydroxyl group relative to the methyl group, but the presence of the phenolic hydroxyl groups makes them electroactive. However, due to their structural similarity, the current-voltage responses of carvacrol and thymol overlap. Therefore, their differentiation using electrochemical methods is difficult. Consequently, when both compounds are present, the concentration is usually reported as the total content of phenolic monoterpenes, which is expressed as either carvacrol or thymol.

Similar to eugenol, there are few bibliographical references dedicated to the application of electrochemical methods for the analysis of carvacrol and thymol in essential oils. Table 2 shows the electrode materials, working conditions, and performance of the voltammetric methods developed for the quantitation of these two isopropylmethylphenols in essential oils and spices. As can be seen, various types of electrodes can be used to fabricate electrochemical sensors. Examples include glassy carbon, platinum, boron-doped diamond, and screen-printed electrodes.

Classical GCE was used to determine the total amount of carvacrol and thymol from black seed oils in a Sörensen-buffered methanol solution using DPV [48], as well as from thyme and oregano essential oils in nonaqueous media (acetonitrile with tetrabutylammonium perchlorate) using LSV [50]. To enhance the analytical detection, SPE modified with SWCNTs [49] was used for the sensitive quantification of isopropylmethylphenols in chemotype Mexican oregano oils. The standard addition method, which is well known for mitigating matrix effects, was used to determine carvacrol and/or thymol in real samples.

Several studies have considered the two compounds individually. Using BDDE, carvacrol was determined in oregano essential oil by DPV, the method being verified through recovery tests using extracts from fresh and dried oregano and marjoram [51]. However, the voltammetric method only allows for the determination of carvacrol in preparations with insignificant amounts of thymol. Stankovic [57] used BDDE in combination with SWV to quantify thymol in *Carum copticum* essential oil.

Another approach to detecting thymol in the essential oil and infusion of *Ocimum gratissimum* (clove basil) used a low-cost electrochemical cell with a pencil lead as the working and counter electrodes [59]. The results obtained by CV and SWV were confirmed by GC-MS.

Carvacrol can be detected and distinguished from thymol in carvacrol-containing essential oils (*Origanum vulgare*, *Thymus vulgaris*, and *Origanum x applii*) exploiting CV and chronoamperometry. This is based on the capacity of carvacrol to form an insulating polymeric layer on the surface of a copper electrode [62]. The detection of carvacrol in the presence of thymol is possible because the thymol does not electropolymerize on the copper surface.

In recent years, some analytical methods that combine electrochemical techniques with chemometric tools have gained significant attention for the quantification of various analytes in complex matrices. In this context, a method for the determination of carvacrol in oregano essential oil was proposed implementing voltammetric second-order modeling based on multivariate curve resolution-alternating least-square (MCR-ALS) [54]. MCR-ALS was applied to the findings obtained by CV, which allowed for the extraction of information from the electrochemical data. This made it possible to assess carvacrol in the presence of thymol.

It has already been pointed out that the overlapping electrochemical responses of thymol and carvacrol complicate their simultaneous determination. However, chemometric approaches can solve this problem. SWV and a platinum microelectrode coupled with partial least squares (PLS) multivariate calibration permitted thymol and carvacrol simultaneous determination in thyme and oregano essential oils [61]. To improve analytical performance, a simple protocol was adopted for preprocessing data obtained by SWV. For thyme assessment, the first derivative and mean-centered were implemented, while mean-centered second derivatives were preferred for carvacrol quantification. In addition to the analytical procedure’s advantages of eliminating the need for previous sample processing and electrode surface modification, it has proven to be environmentally sustainable, meeting the criteria of green analytical chemistry.

Special attention was given to molecularly imprinted polymers (MIPs), synthetic materials that mimic the molecular recognition process of biological macromolecules, such as the interaction between a substrate and an enzyme [63]. In short, MIPs are formed by polymerization of monomers around a model molecule, which is then removed, leaving cavities that bind specifically to the target species. This makes them ideal for creating highly selective electrochemical sensors. Song [60] proposed a double template approach to enable the incorporation of multiple target molecules, such as carvacrol and thymol, during MIP synthesis. The developed sensor—a GCE modified with synthesized MIP—was used as the working electrode to selectively and simultaneously detect carvacrol and thymol in thyme and oregano essential oils. The sensor was produced using tetrabutyl titanate (TBOT) as the functional monomer, 3-aminopropyltriethoxysilane (APTES) as the cross-linker and isopropyl alcohol as the solvent. The detection limits were 0.11 μM for carvacrol and 0.24 μM for thymol. The selectivity of the MIP/GCE was evaluated by investigating the effects of various organic compounds. In the presence of gallic acid, rutin, catechin, quercetin, glucose and urea, the oxidation signal of carvacrol and thymol varied by less than 5%. Although the comparative analysis of the results obtained by LSV and GC-MS showed good agreement, the percentages of carvacrol in oregano essential oil (40.5–48.2%) and thymol in thyme essential oil (55.4–59.4%) represent their mass or volume fraction relative to the total volatile composition of the essential oil.

#### 3.1.3. Other Constituents of Essential Oils

The presence of a phenol group enables the previously mentioned three compounds to participate in redox reactions. Therefore, they are the most extensively studied electrochemically. However, other volatile constituents of essential oils can also undergo electrochemical reactions, and their electroactivity greatly depends on their specific structure and functional groups.

Although certain studies have examined the oxidation or electrochemical behavior of some volatile oil constituents, such as cinnamaldehyde [64], β-caryophyllene [65], α/β-pinene [66] or α-terpinene and α-phellandrene [67], no methods have been developed for their quantitative determination. Table 3 shows the analytical characteristics of the electrochemical methods used to quantify other main components in essential oils and other herbal matrices, namely anethole, limonene, γ-terpinene, menthol, thujone.

Anethole, also known as anise camphor, exists as cis- and trans- isomers, the trans-anethole being the dominant and more stable form. The two isomers have different organoleptic and physicochemical properties. However, cis-isomer is 10–15 times more toxic than the trans-isomer [75]. The electrochemical behavior of trans-anethole is related to its ability to form radical ions at the double bond [69].

To date, only two studies have been reported on anethole quantification. In one investigation several types of electrodes were tested, including GCE, BDDE, and CPE, as well as CPE modified with CeO_2_ and La_2_O_3_, respectively [68]. For quantitative analysis, however, CPE modified with La_2_O_3_ was chosen due to its high sensitivity to anethole, while BDDE was selected for comparison. The BDDE surface was activated for 15 min in 0.1 M H_2_SO_4_ and for 30 s in the supporting electrolyte (0.1 M acetate buffer) before each calibration series. Anethole was detected by DPV in both herbal and commercial matrices, including anise essential oil, which is characterized by high anethole content, and in alcoholic beverages, where it is present at trace concentrations. Non-aqueous CPE is a carbon-based sensor modified with a surfactant soluble in an organic binder that can penetrate the structure of the graphite powder. The presence of this electrochemically inactive surfactant enables DPV measurements to be taken in acetonitrile, considering the low solubility of anethole in aqueous solutions. Results obtained by the developed DPV method are similar to those found using RP-HPLC as reference method.

Limonene exists as two chiral forms, (D)-limonene and (L)-limonene, which are found in varying amounts in citrus fruits and certain pine tree species. Although they share similar chemical properties, the isomers have distinct odors and different applications. (D)-limonene occurs in pharmaceuticals, cosmetics, and personal hygiene products, as well as in solvents. Conversely, (L)-limonene is primarily used in the food industry for flavoring and in cleaning products. An electrochemical sensor based on a nanostructured surface composed of reduced graphene oxide and gold nanoparticles, further modified with electropolymerized polypyrrole, was proposed to selectively determine the enantiomeric compounds [70]. This sensor demonstrated sensitivity and selectivity for both limonene enantiomers, allowing their quantification in orange and pine essential oils.

γ-Terpinene, an isomer of limonene with a different atomic arrangement within the molecule, was quantified in *Bunium persicum* oil using a DPV method with a AuNP-modified GCE [71]. The concentration of γ-terpinene obtained in this manner was similar to the one obtained by GC-MS analysis.

Electrochemical methods have been proposed as a means of analyzing herbal extracts and essential oils to quantify menthol and thujone, which belong to the terpenoid class. Menthol can be determined by DPV using GCE modified with silica microsphere, ferrocene and β-cyclodextrin [72]. Ferrocene can enter into the cavity of β-cyclodextrin to form a stable inclusion complex, while silica improves the modification and enrichment of ferrocene on the electrode surface. The selectivity of the electrochemical sensor was demonstrated in the presence of food additives with a structure similar to that of menthol.

Thujone is a terpenoid ketone existing in two stereoisomeric forms: α-thujone and β-thujone. Although thujone is naturally present in many common plants, large quantities can be toxic to humans. It is known for its potential neurotoxic effects and is associated with substances like absinthe. In addition, α-thujone is more toxic than β-thujone or a mixture of isomers [76]. Kowalcze and Jakubowska [73,74] proposed two electrochemical alternatives for thujone determination in thuja oil and some herbal matrices using DPV. The first approach used a silver amalgam film electrode with HNO_3_ as the supporting electrolyte. The second one used a controlled-growth mercury drop electrode with HNO_3_ or KNO_3_, to which Triton X-100 and ethanol were added. In both cases, thujone’s electrochemical reduction was monitored on the surface of the modified electrodes.

#### 3.1.4. Simultaneous Determination of Volatile Constituents of Essential Oils

The signal overlapping represents the primary challenge of electrochemical techniques in the simultaneous analysis of various components of essential oils due to the structural similarity of their constituents (e.g., thymol and carvacrol).

Although voltammetric techniques have been used successfully to differentiate closely related compounds [77] or even isomers [78], no electrochemical study addresses the simultaneous determination of essential oil constituents. However, two studies quantitatively resolved the overlap of eugenol, carvacrol, and thymol voltammograms in honey samples applying chemometrics.

In the first research, an electroanalytical methodology was developed, based on SWV, GCE and a pH 10 buffer [79]. A multivariate calibration model was defined using an artificial neural network (ANN) for the phenolic compounds. While a pH value of 10 may appear to be elevated for the voltammetric analysis of phenolic compounds such as carvacrol, thymol, and eugenol, it was intentionally employed to ensure the optimal response for these compounds, despite the fact that their oxidation peak currents are approximately half of those attained at pH lower than 8. In the electrochemical analysis of honey using SWV, the ANN method, which is a nonlinear multivariate calibration tool capable of handling complex interactions between analytes and matrix interferences, allowed the deconvolution of overlapping signals corresponding to the three analytes. The findings suggested that the proposed method was applicable, with acceptable recoveries (between 87% and 114%) for all target compounds in honey matrices, with the exception of two samples, whose recovery values were 136% and 72%.

In the second study, a multivariate approach incorporating both DPV on BDDE and partial least-squares (PLS) model allowed the quantitative analysis of eugenol, carvacrol and thymol in honey samples also [80]. The researchers found that creating separate calibration models for each analyte yielded better results than a single multi-analyte model using simple data pre-processing procedures. The proposed method was utilized to ascertain the concentrations of eugenol, carvacrol, and thymol in the ranges of 0.77–13.86, 0.58–10.40, and 0.73–7.32 mg/L, respectively. In comparison with the findings of the preceding study, which employed GCE [79], within the present one the concentration range was twice as wide and the attained detection limits were ten times lower for carvacrol and thymol. The values of some specific parameters, such as the root-mean-squared error of calibration, confirmed the accuracy of the model. Thus, the optimized PLS model was applied for quantification of the three analytes from spiked honey samples, with recovery rates ranging between 80.9% and 111.9%. However, it was possible to effectively identify each of the three compounds when only one of them was present in the sample.

Therefore, combining voltammetric techniques with chemometric methods applied to honey samples could forecast the development of reliable methods for assessing phenolic terpenoids in essential oils. The main challenge arises from the significant differences between the types of matrices being analyzed. While these compounds are found at trace levels in a predominantly aqueous matrix in honey, they are major components in a lipophilic matrix in essential oils. This issue could be tackled using an organic solvent or co-solvent, such as ethanol or methanol.

### 3.2. Electrochemical Assessment of the Antioxidant and Antimicrobial Properties of Essential Oils

The presence of conjugated double bonds and phenolic hydroxyl groups in certain volatile compounds found in essential oils is linked to the oils’ antioxidant properties. These functional groups donate electrons in reaction with radical species, reducing the reactivity of radicals and making them less likely to participate in harmful chain reactions [81]. Moreover, these functional groups can undergo oxidation or reduction at electrodes, making it possible to characterize and screen essential oils using electrochemical methods in order to determine their antioxidant capacity [82]. Specifically, techniques such as CV, DPV, and SWV are used to study the electrochemical properties of both essential oils and their main components. A low oxidation potential, highlighted in the voltammogram during the analysis of a sample, indicates that the compound has a high capacity to donate electrons and, implicitly, a strong antioxidant activity [83]. On the other hand, chronoamperometry, measures the current change over time at a constant potential. This technique can evaluate the antioxidant activity by monitoring the consumption of a specific oxidant or the production of a specific byproduct [84].

Voltammetric can also provide information on the total antioxidant capacity of essential oils, which depends on their chemical composition. This parameter reflects the cumulative effects of all antioxidants present in the matrix, providing a comprehensive overview rather than an individual quantification of each antioxidant [85].

The presence of oxygenated terpenoids and phenylpropanoids in essential oils makes it possible to evaluate their total antioxidant capacity by applying electrochemical methods. Unfortunately, there are not many electrochemical studies related to this topic.

Research on five essential oils from various *Mentha* species confirmed their antioxidant and iron (II) chelation abilities using electrochemical methods [86]. DPV with a platinum electrode was operated to measure the reduction in the limiting current of oxygen electroreduction, which serves as an indicator of the plant extracts’ antioxidant capacity. Another methodology relied on variations in open circuit potential over time to study how these oils chelate ferrous ions. This ability may be linked to the presence of the main compounds pulegone, linalyl acetate, carvone, and l-menthol in each essential oil. *Mentha x gentilis* L. exhibited the highest antioxidant capacity and the fastest rate of Fe^2+^ chelation.

Using SWCNT-SPEs, the voltammetric profiles of two essential oil samples from Mexican oregano, corresponding to wild and cultivated species, revealed a well-defined peak at +0.45 V, which was attributed to the oxidation of the major oil biomarkers, carvacrol and thymol [50]. The differences in peak currents obtained using DPV analysis for the two essential oils enabled discrimination between the plant chemotypes. This was due to the higher carvacrol-thymol content of wild oregano compared to cultivated oregano, as confirmed by GC-MS. Since carvacrol and thymol are indicators of the quality of oregano, the electrochemical index, which is defined as the total amount of antioxidants obtained by electrochemistry [87], was calculated to estimate the total content of biomarkers as carvacrol equivalents. As expected, the wild oregano sample had a higher total biomarker content than the cultivated one.

The antioxidant capacity of 37 essential oil samples from 15 types of plant material was evaluated using the electrooxidation of their antioxidant compounds at the GCE modified with carboxylated MWCNTs [88]. All of the essential oil samples were electrochemically active in a phosphate-buffered solution (pH 7). Well-defined signals were observed on the voltammograms within the 0.0–0.75 V and 0.75–1.5 V ranges, which were due to the oxidation of phenolic antioxidants and terpenoids, respectively. Based on the obtained data, a two-step chronoamperometric method was developed to evaluate the antioxidant capacity of essential oils using anodic potentials of +0.8 and +1.4 V. The authors also compared the data obtained using the traditional DPPH (radical scavenging) and Folin–Ciocalteu (total phenolic content) spectrophotometric assays. Total antioxidant capacity, as determined by chronoamperometric data, showed a significant correlation with DPPH radical scavenging activity, confirming that the results of evaluating antioxidant potential are similar. Furthermore, the significant correlation between the antioxidant capacity determined at +0.8 V and the values obtained by the Folin–Ciocalteu method shows that the electrochemical measurements accurately reflect the total concentration of phenolic compounds. Due to the turbidity generated by the addition of the reagents used in the photometric analysis, the spectrophotometric method could only be applied to a few essential oils—namely clove, cinnamon, nutmeg, and thyme. Among these, thyme oil exhibited the highest antioxidant capacity.

Both the antioxidant and chelating properties of clove and oregano essential oils, as well as their main components, eugenol and carvacrol, were determined by their interaction with Fe(II) ions using CV with a GCE in 0.05 M Na_2_SO_4_ solution [89]. The data pointed out that the electrochemical behavior of essential oils differs slightly from that of their main components, eugenol and carvacrol. The anodic current of Fe(II) decreased by 99.7% in the presence of clove oil, 89.3% for oregano, 99.8% for eugenol, and 99.4% for carvacrol. The difference in peak currents observed between the voltammograms of clove essential oil and eugenol was explained by the presence of (E)-β-ocimene in the essential oil. In the case of oregano essential oil and carvacrol, an opposite effect was observed, which was explained by the fact that only the carvacrol in the essential oil is electroactive on the GCE surface. The study concluded that clove and oregano essential oils, as well as their main constituents, can be used for food preservation due to their antioxidant properties, which involve chelating metal ions. Furthermore, eugenol and carvacrol were more efficient than butylated hydroxytoluene (BHT) standard at inhibiting the formation of secondary lipid peroxidation products in the thiobarbituric acid test (TBARS assay).

The first eco-designed reagent-free paper-based electrochemical sensor was developed for the individual detection of essential oils’ antimicrobials (thymol, eugenol, and carvacrol) testing carbon black as a modifier through the drop casting method [90]. The device was pre-loaded with acetate buffer, requiring the addition of only 5 μL of sample, thus eliminating the need for other reagents or the pH adjustment. The sensor demonstrated a dynamic linear range of up to 16 ppm, with detection limits of 0.1 ppm for thymol and eugenol, and 0.2 ppm for carvacrol. The selectivity of the developed sensor was demonstrated through the evaluation of the DPV signal of carvacrol in the presence of various bacteria and viruses. The accuracy of the sensor was confirmed through a recovery study from seawater samples and a comparison with GC–MS results.

### 3.3. What Electrochemical Methods Can and Cannot Do in Essential Oil Analysis?

GC-FID or GC-MS detection is the logical choice for obtaining a complete phytochemical profile of an essential oil, as it can simultaneously separate and identify (using specialized databases) hundreds of volatile compounds from a single injection. Peak area normalization is used for the semi-quantitative determination of volatile components. This method provides an estimate of the concentration ratio of the target compound to the total concentration of all volatile components in the sample [61,71,82]. To perform an accurate quantitative analysis and determine the actual (nominal) concentration of the analyte, pure reference standards must be used in both GC methods.

On the other hand, electrochemical methods are efficient in terms of analysis time, cost and sample preparation. Samples can be analyzed quickly and easily with minimal pretreatment. Even though essential oils are hydrophobic and insoluble in aqueous media, this limitation can be overcome using simple strategies such as the use of hydro-organic solvent mixtures, surfactants or emulsified systems. The electrochemical methods can also be used for quality control in production lines or for rapidly assessing the total antioxidant activity, free radical-scavenging or redox capacity of a raw essential oil. Furthermore, the portability of electrochemical systems and the single-use electrodes enables testing to be carried out directly at the harvest site or in distillation facilities.

Although the studies cited did not consider the detection of adulteration in essential oils using electrochemical methods, these analytical procedures enable the monitoring of changes in the electrochemical profile of an essential oil arising from dilution, substitution, or the addition of lower-quality constituents. Adulteration often changes the amount of phenolic compounds present, thereby altering antioxidant activity. Consequently, the quantification of electroactive phenols can provide valuable information regarding the authenticity and quality of essential oils. The combination of electrochemical sensing with chemometric tools can enhance the ability to classify samples, identify anomalies, and estimate the degree of adulteration.

One major drawback of electrochemical techniques is that they cannot detect volatile or saturated hydrocarbons (such as non-polar terpenes like limonene and pinene), as these molecules lack electroactive functional groups. Moreover, the complex and highly lipophilic nature of essential oils may hinder electron-transfer processes, cause electrode fouling, and compromise signal quality. Furthermore, while isomers have sometimes been distinguished, identifying certain positional isomers (such as carvacrol or thymol) requires MIP-modified electrodes or chemometric approaches. Although the latter have proven effective in resolving overlapping electrochemical signals, their performance depends on the development and validation of the model. In the studies reported to date, reliable predictive models require appropriate training and external validation to minimize overfitting and ensure satisfactory prediction accuracy. Furthermore, the transferability of models between different essential-oil matrices should be carefully evaluated, since the composition of the matrix may significantly influence predictive performance.

Electrochemical methods cannot fully replace chromatographic techniques for comprehensive compositional analysis, but they offer significant advantages in terms of environmental sustainability, rapid screening, and the development of smart sensing platforms for authenticity assessment in the essential oil industry.

## 4. Conclusions and Perspectives

This review provides insights into current approaches for quantifying the essential oils’ major constituents that exhibit electrochemical activity and discusses key performance characteristics of voltammetric methods reported to date. The potential of electrode surface modification strategies to improve analytical sensitivity and selectivity was highlighted.

As previously outlined, the number of electrochemical methods reported for determining volatile compounds present in significant concentrations in essential oils is relatively limited. This can be attributed to the fact that there are few electroactive compounds in essential oils, as well as to the intrinsic challenges associated with the lipophilic nature of essential oils. Various strategies, including the use of non-aqueous media, co-solvents, surfactants, and polymeric systems, have been pursued to overcome these limitations, although their applicability sometimes depends on the analyte and matrix.

The use of nanomaterials (e.g., graphene, nanotubes, and metal nanoparticles) or polymer films in the modification of electrode surfaces has improved the selectivity and sensitivity of electrochemical sensors toward the target analytes. However, this process can be time-consuming, and the use of certain reagents poses risks to the environment and human health. In this context, researchers must address this dual challenge by offering effective and sustainable alternatives. A first step has been taken with the development of ecological measurement tools such as GAPI (Green Analytical Procedure Indexes), AGREE (Analytical Greenness), or AGSA (Analytical Green Star Analysis), which provide a quantitative assessment of the environmental impact of analytical procedures, addressing different stages of the life cycle, from sample preparation to waste management. Nevertheless, the simultaneous determination of such compounds remains challenging. To address this issue, there has been an increasing tendency to employ various chemometric tools for data processing, facilitating multicomponent determination. However, their application in this field still requires further development, particularly for complex matrices where signal overlap and matrix effects can affect analytical performance. The integration of advanced data-processing and chemometric tools could contribute to improve signal resolution, more reliable analyte discrimination, and enhanced overall analytical performance. Consequently, additional studies are needed to refine method robustness and ensure reliable analytical performance in real-world applications.

Although chromatographic methods are the prevailing standard for separation of complex mixtures of compounds in essential oils and spectrophotometric methods are recognized for efficiently evaluating their antioxidant activity, electrochemical methods have grown in popularity as fast, sensitive, environmentally friendly, and low-cost alternatives. Existing studies suggest that these methods may be useful for estimating antioxidant capacity and determining selected phenolic markers in certain applications. This approach significantly diminishes the need for complex sample preparation, rendering it particularly well-suited for on-site quality control applications.

The information presented in this review will prove useful in various applications, such as the simple determination of the antioxidant capacity of essential oils, the evaluation of their quality by identifying and quantifying specific compounds, but also for detecting adulteration, dilution, or degradation over time. Nevertheless, the performance of electrochemical methodologies and their transferability across different essential oil matrices need in-depth research before broader implementation can be considered. Overall, the available evidence suggests that, rather than replacing established techniques, electrochemical methods represent a promising complementary analytical tool. Future research should focus on improving selectivity and expanding the range of detectable compounds. It should also aim to validate methodologies across diverse matrices and develop more sustainable sensing platforms.

Moreover, in an era where the food industry is undergoing a fundamental shift from reactive testing to instant prediction through artificial intelligence (AI) and internet of things (IoT), electrochemical sensor technology must evolve to create smart devices capable to control food quality with real-time data acquisition, improved efficiency and autonomous decision-making capabilities.

## Figures and Tables

**Table 1:** Electrochemical sensors reported for the detection of eugenol in essential oils and spices.

Electrode and Working Conditions	Detection Technique	Linear Range [μM]	Detection Limit [μM]	Sample	Ref
PVC-graphite electrode; 2.5 mM H_2_SO_4_; E_p_ = +0.3 vs. Ag/AgCl	AMP	0.5–30	0.9	Spices	[32]
GCE; 0.1 M LiClO_4_, 0.1 M Triton X100, ethanol; E_p_ = +0.78 vs. Ag/AgCl	CV	15–1230	3.8	Clove and basil essential oil	[33]
Cu@AuNPs/GCE; BRB (pH 2); E_p_ = + 0.66 V vs. Ag/AgCl	LSV	0.05–0.8 **	0.042 **	Spice and curry powder	[34]
PEDOT:PSS-SWCNTs-PVP/GCE; 0.1 M PBS (pH 6); E_p_~+0.4 vs. SCE	DPV	0.15–122.4	0.048	Curry powder	[35]
MWCNTs-IL/GCE; BRB (pH 10.5); E_p_~+0.2 vs. Ag/AgCl	SWV	0.25–4	0.087	Clove	[36]
MIP/Gr-MWCNTs-IL/GCE; 0.1 M ABS (pH 5); E_p_ = +0.45 vs. SCE	LSV	0.2–20	0.1	Curry powder	[37]
CeO_2_-CPB/GCE; PBS (pH 7); E_p_ = +0.4 vs. Ag/AgCl	DPV	0.075–75	0.0191	Clove, cinnamon, basil and nutmeg essential oils and clove spices	[38]
EUG_(immob.)_-CB-DHP/GCE; 0.2 M PBS (pH 2); E_p_ = +0.5 V vs. Ag/AgCl	SWV	0.029–26 *	0.00013 *	Clove essential oil	[39]
CuSe@rGO/GCE; 0.1 M BRB (pH 2); E_p_~+0.8 V vs. Ag/AgCl	LSV	1–82 ***	0.41 ***	Clove and cinnamon powders	[40]
APTMS-GO@SnO_2_/CPE; 0.1 M PBS (pH 3); E_p_ = +0.65 vs. Ag/AgCl	DPV	0.05–440	0.02	Clove	[41]
PAN-MIP/GPE; 0.1 M PBS (pH 6); E_p_ = +0.45 vs. Ag/AgCl	DPV	0.0005–1, 1–50, 50–160	0.0004	Curry and clove powders	[42]
La_2_Sn_2_O_7_NPs/GCE; 0.05 M PBS (pH 1); E_p_ = +0.78 V vs. Ag/AgCl	DPV	0.01–127, 127–1467	0.006	Clove	[43]
HAP-TiO_2_/GCE; 0.1 M PBS (pH 7.0); E_p_ = +0.51 V vs. Ag/AgCl	SWV	1.4–78	0.094	Clove oil	[44]
CoO-ZnO/GCE; 0.1 M PBS (pH 1); E_p_ = +0.76 V vs. Ag/AgCl	DPV	0.049–179.8	0.004	Clove	[45]
pgABA/GCE; PBS (pH 2.2); E_p_ = +0.654 V vs. Ag/AgCl	CV	0.6–100	0.2	Clove	[46]
polyPGR/MWCNTs-COOH/GCE; BRB (pH 2); E_p_ = +0.57 V vs. Ag/AgCl	DPV	0.75–100	0.73	Clove, cinnamon, and nutmeg essential oils	[47]

* μg; ** μg/mL; *** μg/kg. ABS: acetate buffer; AMP: amperometry; APTMS: aminopropyltrimethoxysilane; BRB: Britton-Robinson buffer; CPB: cetylpyridinium bromide; CPE: carbon paste electrode; CV: cyclic voltammetry; DHP: dihexadecyl hydrogen phosphate; DPV: differential pulse voltammetry; GCE: glassy carbon electrode; Gr: graphene; GO: graphene oxide; GPE: graphite paste electrode; HAP: hydroxyapatite; IL: ionic liquid; LSV: linear sweep voltammetry; MIP: molecularly imprinted polymer; MWCNTs: multi-walled carbon nanotubes; PAN: polyacrylonitrile; PBS: phosphate-buffered solution; PEDOT:PSS: poly(3,4-ethylenedioxythiophene):poly(styrene-sulfonate); pgABA: poly γ-aminobutyric acid; polyPGR: electropolymerized pyrogallol red; PVP: polyvinylpyrrolidone; rGO: reduced graphene oxide; SCE: saturated calomel electrode; SWCNTs: single-walled carbon nanotubes; SWV: square wave voltammetry.

**Table 2:** Electrochemical sensors reported for the detection of carvacrol and thymol in essential oils and spices.

Electrode and Working Conditions	Detection Technique	Linear Range [μM]	Detection Limit [μM]	Sample	Ref
Carvacrol					
GCE; PBS-methanol (pH 8.5); E_p_ = +0.49 V vs. Ag/AgCl	DPV	0.25–2.5 *	0.04 *	Black seed oils	[48]
SWCNT/SPE; PBS (pH 5); E_p_ = +0.5 V vs. Ag/AgCl	DPV	5–50	4	Mexican oregano essential oil	[49]
GCE; ACN, 0.1 M TBAP; E_p_ = +1.28 V vs. Ag/AgCl	LSV	250–1200	86	Oregano and thyme essential oils	[50]
BDDE; 0.1 M ABS (pH 6); E_p_ = +0.945 V vs. Ag/AgCl	DPV	0.29–15.03 *	0.02 *	Oregano essential oil	[51]
PtE; CH_3_COOH, ACN, 0.1 M NaClO_4_; E_p_ = +1.190 V vs. Ag/AgCl	DPV	0.47–640 *	0.05 *	Herbal spices	[52]
MWCNT/GCE; PBS (pH 6.5); E_p_ = +0.524 V vs. Ag/AgCl	DPV	0.1–25; 25–150	0.075	Oregano	[53]
GCE; ACN, 0.1 M TBAP; E_p_ = +1.28 V vs. Ag/AgCl	CV	104–1560	62.7	Oregano essential oil	[54]
MWCNT-TP/GCE; BRB (pH 2); E_p_ = +0.83 V vs. Ag/AgCl	DPV	0.1–10; 10–100	0.063	Herbal spices	[55]
Thymol					
GCE; PBS-methanol (pH 8.5); E_p_ = +0.49 V vs. Ag/AgCl	DPV	0.25–2.5 *	0.04 *	Black seed oils	[48]
SWCNT/SPE; PBS (pH 5); E_p_ = +0.5 V vs. Ag/AgCl	DPV	5–90	4	Mexican oregano essential oil	[49]
GO/GCE; 0.2 M BRB (pH 4.7); E_p_~+0.6 V vs. Ag/AgCl	DPV	2–200	0.065	Thyme plant	[56]
BDDE; BRB (pH 4); E_p_ = +1.13 V vs. Ag/AgCl	SWV	4–20; 20–100	3.9	Ajowan essential oil	[57]
CeO_2_-Brij^®^35/GCE; PBS (pH 6); E_p_ = +0.59 V vs. Ag/AgCl	DPV	0.7–10.1; 10.1–606	0.2	Oregano	[58]
GCE; ACN, 0.1 M TBAP; E_p_ = +1.29 V vs. Ag/AgCl	LSV	220–1300	79	Oregano and thyme essential oils	[50]
PtE; CH_3_COOH, ACN, 0.1 M NaClO_4_; E_p_ = +1.195 V vs. Ag/AgCl	DPV	0.39–1105 *	0.04 *	Herbal spices	[52]
MWCNT-TP/GCE; BRB (pH 2); E_p_ = +0.81 V vs. Ag/AgCl	DPV	0.05–25; 25–100	0.037	Herbal spices	[55]
GCE and PGE; 0.5 M H_2_SO_4_/ethanol; E_p_ = +0.97 V vs. Ag/AgCl	SWV	50–200	-	Clove basil essential oil	[59]
Carvacrol and thymol simultaneously					
MIP/GCE, ABS (pH 5); E_carvacrol_ = +1.14 V; E_thymol_ = +0.92 V vs. SCE	LSV	carvacrol: 0.8–50 thymol: 1–50	carvacrol: 0.11 thymol: 0.24	Oregano and thyme essential oils	[60]
PtE; ACN, 0.1 M TBAP; E_carvacrol_ = +1.74 V; E_thymol_ = +1.52 V vs. Ag/AgCl	SWV	carvacrol: 93–1420 thymol: 92–1380	carvacrol: 9.8 thymol: 7.4	Oregano and thyme essential oils	[61]

* μg/mL. ABS: acetate buffer; ACN: acetonitrile; BDDE: boron-doped diamond electrode; BRB: Britton-Robinson buffer; CV: cyclic voltammetry; DPV: differential pulse voltammetry; GCE: glassy carbon electrode; GO: graphene oxide; LSV: linear sweep voltammetry; MIP: molecularly imprinted polymer; MWCNT: multi-walled carbon nanotubes; PBS: phosphate-buffered solution; PGE: pencil graphite electrode; PtE: platinum electrode; SPE: screen-printed electrode; SWCNT: single-walled carbon nanotubes; SWV: square wave voltammetry; TBAP: tetrabutylammonium perchlorate.

**Table 3:** Electrochemical sensors reported for the detection of other main constituents of essential oils.

Electrode and Working Conditions	Detection Technique	Linear Range [μM]	Detection Limit [μM]	Sample	Ref
Anethole					
BDDE; 0.1 M ABS (pH 6); E_p_ = +0.965 V vs. Ag/AgCl	DPV	0.7–17.5 *	0.024 *	Herbals, anise essential oil	[68]
GCE; 0.1 M ABS (pH 6); E_p_ = +0.990 V vs. Ag/AgCl	DPV	0.7–17.5 *	0.011 *	-	[68]
CPE; 0.1 M ABS (pH 6); E_p_ = +1.155 V vs. Ag/AgCl	DPV	0.7–17.5 *	0.006 *	-	[68]
La_2_O_3_/CPE; 0.1 M ABS (pH 6); E_p_ = +1.095 V vs. Ag/AgCl	DPV	0.7–17.5 *	0.04 *	Herbals, anise essential oil	[68]
SDS/CPE; ACN, 0.1 M LiClO_4_; E_p_ = +1.289 V vs. SCE	DPV	2–200	0.7	Anise and fennel seeds	[69]
D-Limonene					
rGO-AuNPs-PPy/GCE; 0.1 M PBS (pH 7); E_p_~+0.25 V vs. Ag/AgCl	DPV	2·10^−6^–1·10^−5^; 1·10^−5^–1·10^−4^	1.8·10^−6^	Mint and essential oil	[70]
L-Limonene					
rGO-AuNPs-PPy/GCE; 0.1 M PBS (pH 7); E_p_~+0.25 V vs. Ag/AgCl	DPV	2·10^−6^–1·10^−5^; 1·10^−5^–1·10^−4^	4·10^−6^	Mint and essential oil	[70]
γ-Terpinene					
AuNPs/GCE; ACN, 0.1 M LiClO_4_; E_p_ = +1.5 V vs. Ag/AgCl	DPV	100–12,000	0.5	Black cumin oil	[71]
Menthol					
SiO2@Fc-CD/GCE; 0.1 M KCl; E_p_~+0.45 V vs. Ag/AgCl	DPV	0.025–0.1	0.01636	Mint and essential oil	[72]
Thujone					
Hg(Ag)FE; 0.0115 M HNO_3_ (pH 1.85), ethanol; E_p_ = −0.22 to −0.126 V vs. Ag/AgCl	DPV	0.7–142 *	0.05 *	Herbal matrices, thuja oils	[73]
CGMDE; 0.04 M H_3_PO_4_ (pH = 1.9) or 0.05 M KNO_3_ (pH = 6.4), Triton X-100, ethanol; E_p_ = −0.005 V (H_3_PO_4_); E_p_ = −0.109 V (KNO_3_) vs. Ag/AgCl	DPV	0.7–7; 7–140 * (H_3_PO_4_) 0.7–115 * (KNO_3_)	0.4 * (H_3_PO_4_) 0.6 * (KNO_3_)	Herbal matrices, thuja oils	[74]

* μg/mL. ABS: acetate buffer; ACN: acetonitrile; AuNPs: gold nanoparticles; BDDE: boron-doped diamond electrode; CD: cyclodextrin; CGMDE: controlled growth mercury drop electrode; CPE: carbon paste electrode; DPV: differential pulse voltammetry; Fc: ferrocene; GCE: glassy carbon electrode; Hg(Ag)FE: renewable silver amalgam film electrode; PPy: poly-pyrrole; rGO: reduced graphene oxide; SCE: saturated calomel electrode; SDS: sodium dodecyl sulfate.

## Data Availability

No new data were created or analyzed in this study. Data sharing is not applicable to this article.
